# Preclinical evaluation of sentinel node localization in the stomach via mannose-labelled magnetic nanoparticles and indocyanine green

**DOI:** 10.1007/s00464-023-10099-6

**Published:** 2023-05-10

**Authors:** Aidan Cousins, Shridhar Krishnan, Giri Krishnan, Nguyen Pham, Valentina Milanova, Melanie Nelson, Anil Shetty, Naruhiko Ikoma, Benjamin Thierry

**Affiliations:** 1 Future Industries Institute, University of South Australia, Mawson Lakes Campus, Adelaide, SA 5095 Australia; 2 Department of Oral and Maxillofacial Surgery, The University of Adelaide, Adelaide, SA 5000 Australia; 3 Department of Otolaryngology, Head and Neck Surgery, The University of Adelaide, Adelaide, SA 5000 Australia; 4 Key Centre for Polymers and Colloids, School of Chemistry and University of Sydney Nano Institute, The University of Sydney, Sydney, NSW 2006 Australia; 5 Ferronova Pty Ltd, MM-Building, Minerals Lane, Mawson Lakes, SA 5095 Australia; 6 Department of Surgical Oncology, The University of Texas MD Anderson Cancer Center, Houston, TX USA

**Keywords:** Gastric cancer, Sentinel lymph node, Magnetic nanoparticles, Indocyanine green, Dual modality, MRI

## Abstract

**Background:**

Gastrectomy with extended (D2) lymphadenectomy is considered standard of care for gastric cancer to provide the best possible outcomes and pathologic staging. However, D2 gastrectomy is a technically demanding operation and reported to be associated with increased complications and mortality. Application of sentinel lymph node (SLN) concept in gastric cancer has the potential to reduce patient morbidity; however, SLN techniques are not established for gastrectomy, in part due to lack of practical tracers. An effective and convenient tracer with enhanced SLN accumulation is critically needed.

**Methods:**

Mannose-labelled magnetic tracer ‘FerroTrace’ and fluorescent dye indocyanine green (ICG) were injected laparoscopically into the stomach submucosa of 8 healthy swine under general anaesthesia. Intraoperative fluorescence imaging was used to highlight draining lymphatic pathways containing ICG, while preoperative T2-weighted MRI and ex vivo magnetometer probe measurements were used to identify nodes containing FerroTrace. Lymphadenectomy was performed either robotically (*n* = 2) or via laparotomy (*n* = 6).

**Results:**

Mixing ICG and FerroTrace ensured concurrence of fluorescent and magnetic signals in SLNs. An initial trial with robotic dissection removed all magnetic LNs (*n* = 4). In the subsequent laparotomy study that targeted all ICG-LNs based on intraoperative fluorescence imaging, dissection removed an average of 4.7 ± 1.2 fluorescent, and 2.0 ± 1.3 magnetic LNs per animal. Both MRI and magnetometer detected 100% of SLNs (*n* = 7). FerroTrace demonstrated high specificity to SLNs, which contained 76 ± 30% of total lymphotropic iron, and 88 ± 20 % of the overall magnetometer signal.

**Conclusions:**

Through utilisation of this dual tracer approach, SLNs were identified via preoperative MRI, visualised intraoperatively with fluorescence imaging, and confirmed with a magnetometer. This combination pairs the sensitivity of ICG with SLN-specific FerroTrace and can be used for reliable SLN detection in gastric cancer, with potential applications in neoadjuvant therapy.

**Graphical abstract:**

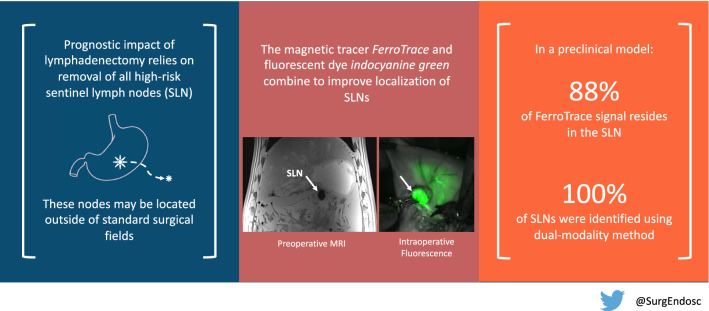

The sentinel lymph node (SLN) concept—whereby a conservative lymphadenectomy prioritises the first lymph nodes (LN) to receive drainage from a primary tumour—has been broadly and successfully applied to a multitude of cancers since technical feasibility of this approach was reported by Morton et al. [1]. The SLN concept has transformed the surgical approach in which breast cancer and melanoma are routinely treated, and created a new ‘gold standard’ over more invasive procedures (such as axillary LN dissection in breast cancer). By limiting the number of resected LNs, SLN biopsy (SLNB) reduces the need for more aggressive lymphatic dissection in cN0 patients, thereby reducing patient morbidity, simplifying surgical dissection and workflow, and enabling more detailed pathological examination of fewer removed LNs.

For stomach cancer, the third global leading cause of death among cancers [2], gastrectomy with extended (D2) lymphadenectomy is considered standard of care due to the high incidence of metastases at regional LNs of the disease [3]. However, in patients without LN metastases (N0), standard D2 lymphadenectomy can be considered overtreatment, and may often lead to unnecessary morbidity and mortality [4]. In these patients, a much more specific and targeted approach to staging LN metastases could be beneficial [4–6], thereby creating a role for an accurate means of SLN localization in gastric cancer prognosis and treatment. While SLNB concepts, such as the conservative ‘pick-up’ method of lymphadenectomy have been trialled in gastric cancer [4], given the complexity of lymphatic drainage in this region, improved prognostic outcomes are correlated with an increased number of removed LNs [4, 7, 8]. Here, a greater number of removed LNs increases the chance of capturing the high-risk SLNs likely to contain metastases. As such, while SLNB is technically feasible in gastric cancer [9, 10], the pick-up method appears less effective at capturing high-risk LNs compared to more extensive approaches, such as sentinel node navigation surgery (SNNS) [4, 11]. In the latter approach, lymphotropic tracers such as indocyanine green (ICG) dye or the combination of radiocolloid and blue dye tracers are used to help define the surgical field in which these high-risk nodes are most likely to be located [10–12]. This is especially helpful when these prognostically significant LNs are located in distant sites and would otherwise not be removed by routine lymphadenectomy [8]. Following lymphadenectomy, the whole specimen is analysed by pathology to identify metastases within the harvested LNs [11].

Ultimately, successful application of the SLN concept to any cancer relies on accurately identifying the SLN as distinct from higher echelon LNs. Indeed a recent meta-analysis of SLNB procedures in gastric cancer concludes that both ICG and the radiocolloid/blue dye combination are acceptable means of mapping the lymphatics [10]. However, these approaches are not without their limitations. Despite identifying relevant lymphatic pathways, ICG and blue dyes are non-specific, flow rapidly through multiple LN echelons, and are unable to define the SLN as distinct from higher echelon LNs. Conversely, radiocolloid tracers migrate more slowly and accumulate in fewer LNs compared to ICG [10], but require careful handling and access to specialised manufacturing facilities. Furthermore, high concentrations of radiocolloid tracer at the injection site produces ‘shine-through’ radiation that can obscure the SLN signal when in close proximity to the primary tumour [9, 13–15]. This is to say nothing of the additional health risks and hazards associated with radioactive substances, and the training and licencing required for correct transport, administration, storage, and disposal. Given the importance of accurate identification of the SLN, an effective and convenient tracer for gastric cancer that can overcome the above limitations is critically needed.

To this end, we propose a dual-modality technique for gastric SLN localization, utilizing a combination of magnetic and fluorescent agents. As with the combination of radiocolloids and blue dyes, we aim to pair two complementary modalities in order to take advantage of the strengths of each, while overcoming the limitations of individual tracers. A novel mannose-labelled iron oxide nanoparticle ‘FerroTrace’ (Ferronova, Adelaide, SA Australia) has previously demonstrated efficacy as a lymphotropic magnetic tracer in the swine head and neck, given the strong signal contrast created in MRI allowing preoperative mapping, and its high specificity to SLNs as a result of macrophage binding between the CD206 receptor and the tracer’s mannose label [16, 17]. While FerroTrace is capable of differentiating SLNs (based on magnetic signals measured by a handheld magnetometer probe [16]), it is not easily visualised or detected in vivo in the gastric environment. On the contrary, fluorescent ICG is excellent at highlighting draining lymphatic pathways in vivo, despite its poor specificity to SLNs. As such, a combination of FerroTrace and ICG creates a means of lymphatic mapping whereby preoperative MRI and intraoperative fluorescence imaging can be used to define the surgical field for lymphadenectomy, and a magnetometer probe can then be used to accurately localize the SLN among the multitude of dissected LNs.

In this study, the combination of FerroTrace and ICG is applied to a large animal stomach model in order to determine the efficacy of this dual-modality approach and propose a workflow model designed for gastric cancer patients in the hope of facilitating wider application of targeted lymphadenectomy in this region.

## Materials and methods

### Animal details

All animal experiments were performed according to a protocol approved by the Animal Ethics Committee of the South Australian Health and Medical Research Institute (SAHMRI). Animal work was performed at SAHMRI facilities in compliance with The Australian Code for the Care and Use of Animals for Scientific Purposes, 8th edition, 2013; and The South Australian Animal Welfare Act, 1985. Eight female domesticated pigs (*sus scrofus;* Large White × Landrace) were used for this study with a mean body weight of 54 ± 6 kg. Prior to any interventions, animals were placed under general anaesthesia via intramuscular injection of ketamine (10 mg/kg), followed by intubated delivery of isoflurane and oxygen (5% initial, 2% maintenance). Animals were humanely euthanized prior to dissection via overdose of intravenous pentabarbitone.

### Tracer preparation

The magnetic tracer ‘FerroTrace’ was supplied by the manufacturer (Ferronova, Adelaide, SA, Australia) as a circa 55 mg/mL sterile suspension in 0.9% saline. ICG (Cardiogreen, Sigma Aldrich, St Louis, MO, USA) was purchased in bulk as a dried powder, and separated into smaller aliquots of 2 mg each. ICG dye solution was prepared no more than 1 h before injection by mixing the powder with sterile water for injection to a concentration of 2.5 mg/mL. Immediately prior to injection, a ‘combination’ tracer was prepared by mixing the desired ratios of ICG and FerroTrace in a separate vial. All preparation involving ICG occurred at room temperature, shielded from ambient light.

An injection needle set-up was constructed by connecting a short 23G needle to a 75 cm extension set (1.3 mL priming volume) and 3-way valve. To the valve were connected a syringe containing the combination tracer, and a syringe containing isotonic saline. Prior to injection, the needle was primed with the combination tracer, after which the valve was switched and saline was used to flush through combination tracer to control delivery of the required dose.

### MRI scans

A 3.0 T Magnetom Skyra MRI scanner (Seimens-Healthineers, Erlangen, Germany) was used to image the swine abdomen in order to preoperatively identify magnetic-positive LNs. FerroTrace particles are highly magnetically susceptible, and create strong negative contrast on MRI scans, even at low concentrations. For this study, two protocols were used, a T2-weighted scan (2D GRE FLASH, TE = 4.27 ms; TR = 540 ms; FA = 20°; 2 mm slices), and a T1-weighted volumetric interpolated breath-hold examination (VIBE) scan (3D multi-echo, TE = 1.33, 3.79, 6.25, 8.71, 11.17 ms; TR = 13.21 ms; FA = 9°; 3 mm slices).

### Robotic pilot study

At the onset of this preclinical investigation, a da Vinci Xi robotic system (Intuitive Surgical Inc., Sunnyvale, CA, USA) was utilised to perform the tracer injection and lymphatic dissection on 2 animals. A dose of 0.25 mL combination tracer (0.15 mL FerroTrace and 0.1 mL ICG dye) was used in both animals, with the objective of initially assessing the feasibility of ICG-guided surgical dissection of gastric LNs—as had been demonstrated previously in the head and neck [17].

**Fig. 1:**
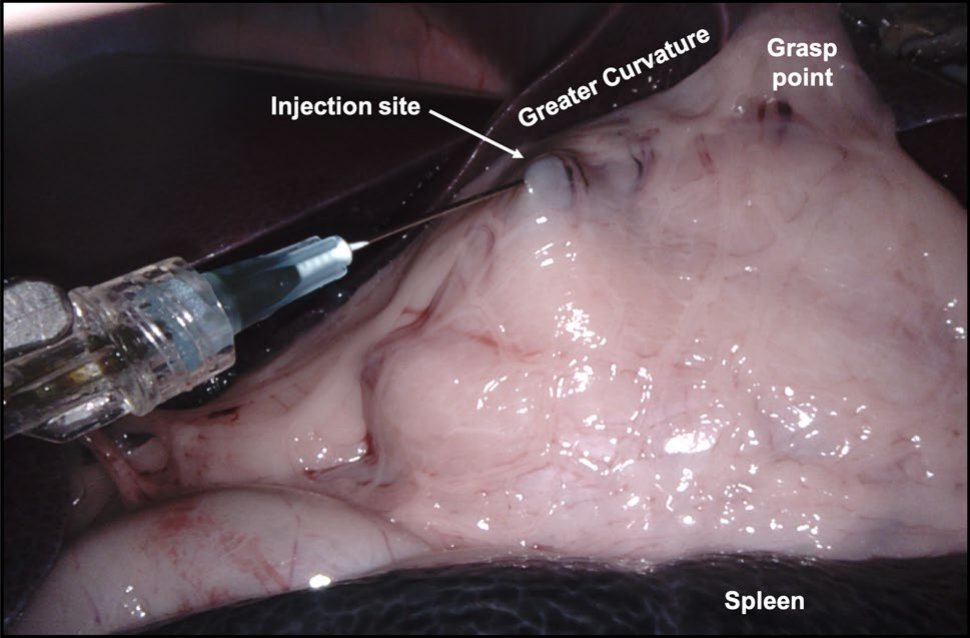
Laparoscopic view of an injection into the greater curvature of a swine stomach using the da Vinci Xi robot

Animals were placed in a supine position on an operating theatre table, and four 10 mm trocar ports were placed into the abdomen to allow access for 2 robot accessories (30° endoscope and Maryland bipolar forceps), a pair of handheld laparoscopic forceps, and the injection needle. An injection site at the posterior distal stomach, close to the greater curvature was chosen (Fig. 1). This site was anticipated to create lymphatic drainage to infra-pyloric gastric nodes [18], which was considered to be easy to access during dissection.

After insufflation of the abdomen with CO_2_ gas, the injection needle was placed (while capped) into the abdomen. Robotic forceps were used to grasp and position the needle, while handheld forceps were used to de-cap the needle and to position the stomach for injection. The combination tracer was delivered in a single, 0.25 mL bolus into the stomach submucosa. Approximately 30 min after injection, animals were scanned with MRI to identify draining LNs containing the magnetic tracer before returning to theatre for dissection. Dissection was performed post mortem, and guided by the da Vinci Xi’s ‘Firefly’ camera in both bright-field and fluorescence imaging modes. Fluorescent lymphatic vessels were followed in an inferior direction from the injection site to draining LNs. Once all magnetic LNs identified on MRI had been resected, the procedure was concluded.

### Laparotomy study

Following the pilot robotic study, the protocol was adjusted to promote improved tracer uptake and identification of nodes by increasing tracer dose and modifying the method of dissection. In these animals (*n* = 6), the injected volume was increased to a single 0.5 mL bolus, consisting of 0.25 mL FerroTrace and 0.25 mL ICG. The robot was replaced in favour of handheld tools in order to achieve a faster, more accessible and comprehensive dissection of draining LNs. After placement of trocars (two 12mm and two 5 mm) and abdominal insufflation with CO_2_ gas, a laparoscopic fluorescence imaging camera (Karl Storz Image1 S ICG camera; RUBINA light source; 10 mm, 0° laparoscope; Karl Storz—Endoskope, Tuttlingen, Germany) was used to guide handheld laparoscopic forceps and the injection needle placement. As with the robotic pilot study animals, the combination tracer was injected into the stomach submucosa, on the right side of the stomach, close to the greater curvature.

One hour after injection, animals were humanely euthanised, after which the superior abdomen was scanned with MRI to localise FerroTrace-positive nodes and help plan and guide surgery. In theatre, a large incision was placed across the abdomen inferior to the diaphragm, to facilitate a more thorough LN dissection through exploratory laparotomy guided by fluorescent ICG. Here, all fluorescent LNs within the draining basin were removed, regardless of the number of magnetic LNs identified on MRI.

### Measurements of magnetic signal and iron level in dissected LNs

Ex vivo*,* each resected LN was measured with an experimental handheld magnetic-sensing (magnetometer) probe (FerroMag, Ferronova, Adelaide, SA, Australia). For each animal, the magnetometer probe was used to determine which resected LNs contained FerroTrace (registered a positive magnetic signal of any magnitude). All dissected LNs were measured using the magnetometer, and a semi-quantitative signal recorded for each. For each animal, SLNs were identified as the LNs with the highest magnetic signal [16, 17] and which received direct lymphatic flow from the injection site (determined via fluorescence imaging). In instances where there was bilateral drainage, a SLN was identified for each side. LNs receiving lymphatic flow-through of ICG or FerroTrace from a SLN were considered to be 2nd echelon.

Following probe measurement, LNs dissected as part of the exploratory laparotomy study were tested for total iron content via inductively coupled plasma mass spectrometry (ICP-MS), following complete digestion of the samples in a mixture of ultrapure concentrated nitric acid and hydrochloric acid.

Where appropriate, statistical calculations (mean, standard deviation, *p* value) were calculated in MATLAB (MathWorks, Natick, MA, USA) using built-in functions.

## Results

### Preoperative MRI

**Fig. 2:**
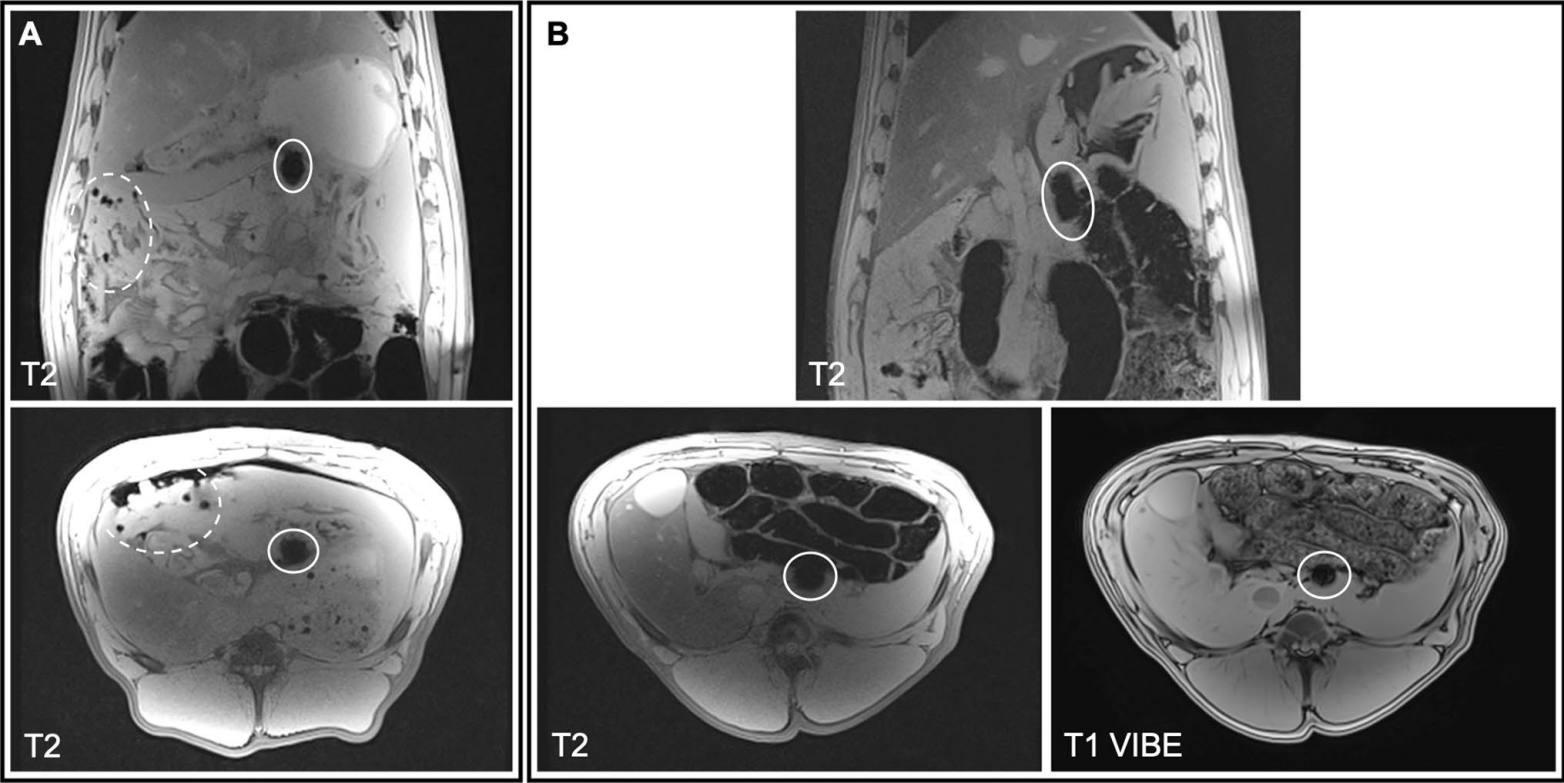
MRI scans of swine abdomen coronal (top) and axial (bottom) planes were used to locate draining LNs (solid oval). **A** An example T2 scan where a magnetic LN is clearly distinguishable from air pockets (dashed oval), surrounding tissue, and large bowel. **B** An example where LN mapping using the T2 protocol was made difficult by LN proximity to the large bowel. In this instance the T1 VIBE protocol (1.33 ms TE) was more effective at distinguishing the SLN

The default protocol for identifying nodes containing the FerroTrace tracer on preoperative MRI was the T2-weighted coronal plane scan. A total of 8 animals underwent MRI imaging for evaluation of preoperative LN mapping. On this scan, FerroTrace at the injection site and inside draining LNs produced very strong negative contrast and susceptibility ‘bloom’ artefact in T2-weighted MRI (Fig. 2). However, in all animals there existed some form of artefact mimicking the negative contrast of FerroTrace-positive LNs as a result of gas pockets (e.g. among the small bowel), introduced when the abdomen was insufflated for injection. Likewise, in all but 1 animal (animal 3), the large bowel—which produced endogenous negative contrast on the T2-weighted scan—was positioned in close proximity to the stomach and overlapped regions where draining LNs were anticipated to reside (Fig. 2). Fortunately, the positioning of the bowel only impacted the identification of FerroTrace-positive LNs in 1 instance (animal 5), whereby the bowel obscured the signal of a single LN on T2-weighted MRI. In this instance, positive MRI identification was instead achieved using the T1 VIBE protocol, which was able to differentiate between signals from the bowel and FerroTrace, particularly when using the shortest TE (1.33 ms) in the multi-echo series (Fig. 2). Despite this success, the T1 VIBE protocol results in a significant reduction in the characteristic signal from FerroTrace; hence the T2-weighted protocol remained the default for preoperative LN mapping.

### Robotic-assisted lymphadenectomy

**Fig. 3:**
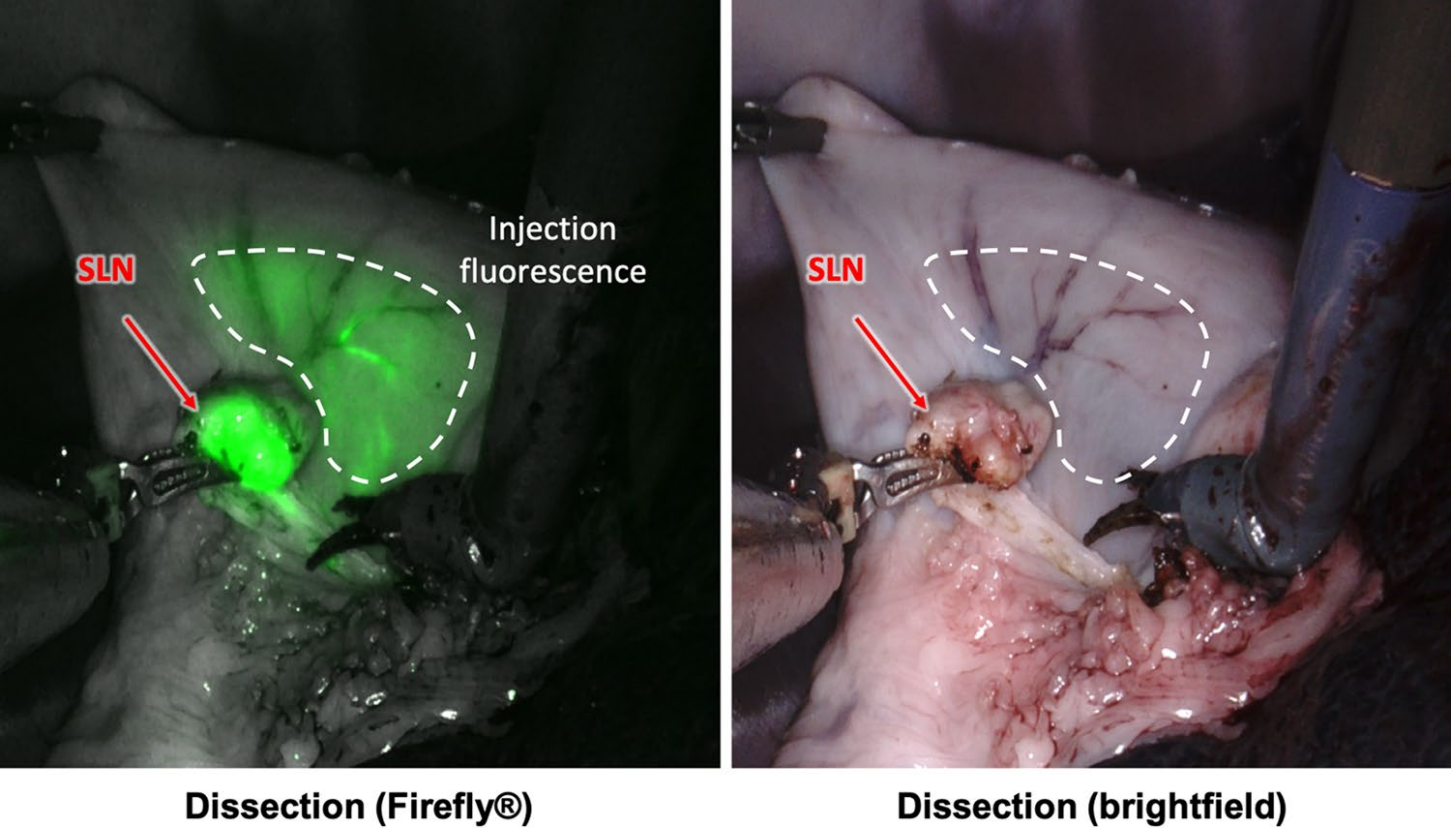
Intraoperative comparison of the fluorescence-imaging guidance and brightfield imaging using the da Vinci Firefly camera. The sensitive fluorescence-imaging of indocyanine green (ICG) dye reveals efferent vessels draining from the submucosal injection point into a nearby gastric sentinel lymph node (SLN)

During surgery, robotic dissection was successfully guided by Firefly fluorescence imaging, whereby the lymphatic vessels efferent to the injection site were clearly identified in both animals and could be followed down to the draining LNs (Fig. 3). Two LNs were removed from both animals, based on the anatomic information provided by preoperative MRI. In this regard, the robotic lymphadenectomy was more akin to the pick-up method of SLNB. After robotic dissection, the magnetometer measured a positive magnetic signal in 3 (75%) of the 4 dissected LNs. As the SLN was defined for each animal based on the LN with the highest signal strength, in the instance where only 1 LN registered a positive magnetometer signal, it was automatically granted ‘SLN’ status. All excised LNs were placed into sample containers with 10% formalin solution, and re-imaged with MRI. These ex vivo scans confirmed that all 4 LNs (100%) were positive for magnetic tracer; hence the false-negative magnetometer detection was likely the result of FerroTrace uptake below the magnetometer’s detection limit.

### Laparotomic lymphadenectomy

**Fig. 4:**
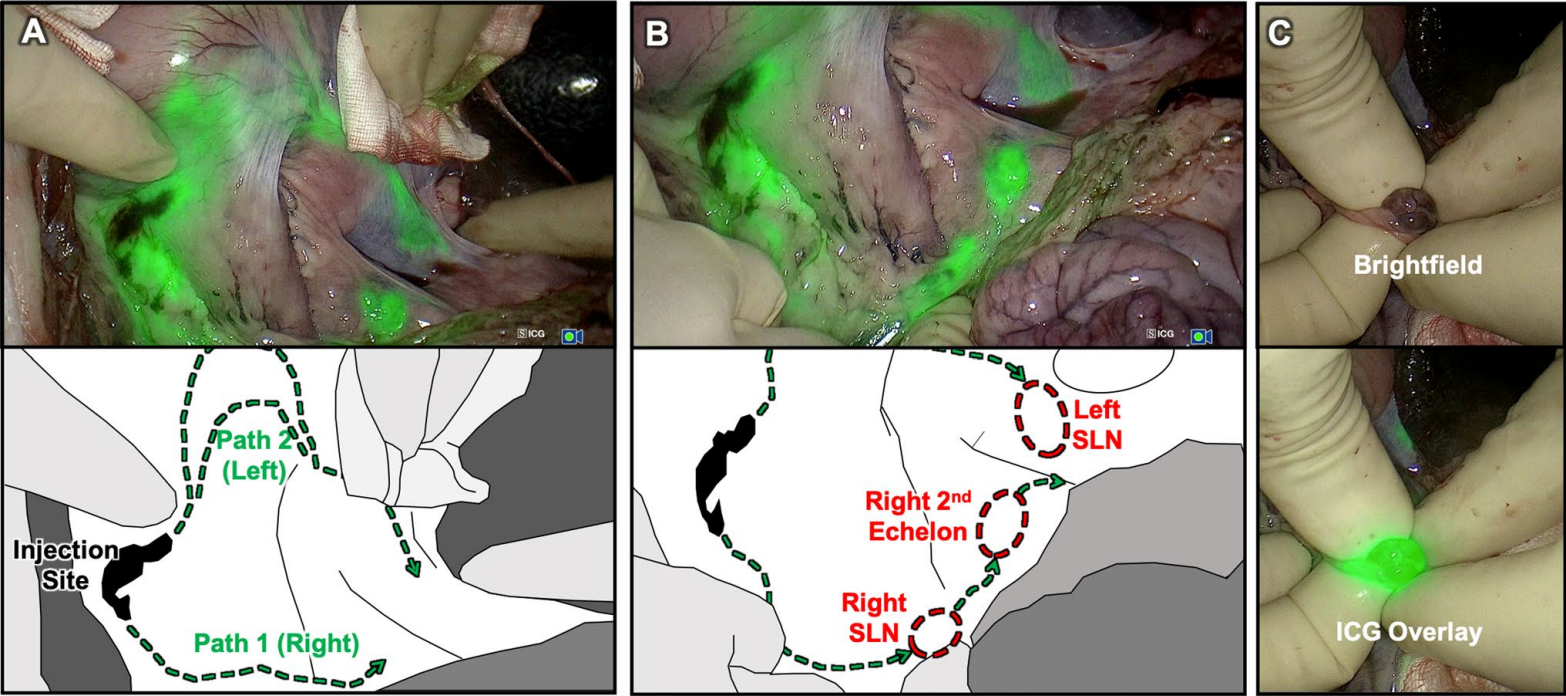
**A** Identification of the draining vessels leading from the injection site—in this case there was bilateral drainage.** B** Following the vessels to locoregional LNs, and in this case revealing a concealed Right SLN. **C** Confirmation that the LN removed (Right SLN) was ICG-positive; in this case, the LN also presented a visible dark discolouration due to the uptake of FerroTrace (Color figure online)

Immediately after injection of the FerroTrace-ICG combination, lymphatic uptake was visible on the laparoscopic fluorescence camera. Monitoring the ICG signal for several minutes post-injection allowed confirmation of the injection, as well as reveal the general direction of lymphatic flow. Animals in this study were euthanized after a median time of 60 min post-injection (range 56–61 min) in an attempt to control the extent of lymphatic uptake before post-mortem MRI and surgical dissection. During dissection, real-time fluorescence imaging of the lymphatic flow appeared a reliable method for locating draining LNs. In this regard, the laparotomic lymphadenectomy was more akin to the SNNS approach of SLNB. The mean ± standard deviation time to locate and remove the first ICG-positive LN using the fluorescence camera was 2.5 ± 1.7 min, and total time to dissect all ICG-positive LNs was 17.8 ± 3.0 min. Figure 4 demonstrates the general approach taken, whereby the fluorescence camera was used to map lymphatic vessels draining from the injection site into LNs, and to confirm positive ICG uptake into LNs once isolated.

In total, 28 ICG-positive LNs were removed with 4.7 ± 1.2 LNs per animal (Table 1). Of these, 11 LNs were FerroTrace-positive, as identified by preoperative MRI (1.8 ± 1.3 per animal). Ex vivo measurement of LNs with the magnetometer probe was less sensitive than MRI, with a total of 9 (6 SLN, 3 higher echelon) FerroTrace-positive LNs detected (1.5 ± 1.2 LNs per animal; Table 1).

**Table 1:** Summary of the number of LNs detected for each laparotomy animal using different modalities

Animal	Number of SLNs	ICG-positive LNs	FerroTrace-positive LNs	MRI-positive LNs	Magnetometer-positive LNs
1	1	4	3	3	3
2	2	6	4	4	3
3	1	6	2	1	0
4	1	5	1	1	1
5	1	4	1	1	1
6	1	3	1	1	1
Mean^a^	1.2 ± 0.4	4.7 ± 1.2	2.0 ± 1.3	1.8 ± 1.3	1.5 ± 1.2

*LN* lymph node, *SLN* sentinel lymph node

^a^Mean value ± standard deviation

In order to more accurately determine the number of FerroTrace-positive LNs (beyond in vivo MRI detection), the total iron content was measured via ICP-MS for each fluorescent LN. Based on measurements of control gastric nodes (FerroTrace-negative), the mean level of endogenous iron in this environment was 12 ± 14 µg. LNs were considered FerroTrace-iron-positive if the measured iron level was above 40 µg (mean level + 2$\sigma$), and ≥ 10% of the maximum iron level in that basin (emulating the inclusive ‘10% rule’ applied to SLNB using conventional radiotracers [19]). Using this method of classification, there were a total of 12 FerroTrace-iron-positive LNs, with a mean of 2.0 ± 1.3 per animal (Table 1).

With this iron quantification data, it was also possible to determine the mean injected dose (ID) accumulation in the LNs. Based on an ID of circa 14 mg iron and using the iron quantification data, the mean FerroTrace accumulation in SLNs (at *t* = 60 min) was calculated to be 3.6 ± 1.9 %ID per animal. The mean total lymphotropic uptake of FerroTrace (measured as cumulative iron in all dissected LNs) was calculated to be 4.8 ± 2.7 %ID per animal.

Given the variation of total FerroTrace-iron uptake (%ID) between animals, a more appropriate measure of tracer distribution is to normalise the FerroTrace-iron content of each LN to the total lymphotropic FerroTrace-iron level on a per animal basis. This analysis produced a mean FerroTrace distribution of 76 ± 30% uptake into the SLNs, with 24 ± 5 % distributed among the higher echelon LNs, which represents a statistically significant difference (2-variable *t* test* p* value = 0.0014, $\alpha$ = 0.05). Similarly, the magnetometer signal for each positively identified LN can be normalised to the total signal per animal, resulting in a signal distribution of 88 ± 20% in SLNs and 24 ± 25% in the higher echelon LNs, again a statistically significant difference (*p* value = 0.032).

## Discussion

For stomach cancer, the classification used to define the extent of lymphadenectomy is based on the location of LN stations removed, as originally defined by the Japanese Research Society for Gastric Cancer [20]. For example, for total gastrectomy, D1 lymphadenectomy generically involves removing LN stations 1–7, while D2 lymphadenectomy involves removal of stations 1–12a [3, 21]. Completion of D2 lymphadenectomy may require pancreatectomy and/or splenectomy [3]. As a result of this more invasive dissection compared to D1, D2 lymphadenectomy results in higher morbidity (D1, 25%; D2, 43%) and mortality (D1, 4%; D2, 10%) [22], and carries with it high instances of bleeding, pancreatitis, subdiaphragmatic abscess, lymphorrhea, and chylous ascites [13]. Despite the more thorough dissection in D2 lymphadenectomy, a Dutch randomised clinical trial [23] found that during patient follow-up the impact of these morbidities associated with surgery resulted in no significant improvement in 5-year survival rate compared to D1 lymphadenectomy (D1, 45%; D2, 47%). However, in the longer term there was a 15-year survival rate benefit for D2 as a result of lower rates of locoregional recurrence (D1, 22%; D2, 12%) due to a more thorough removal of metastatic LNs in D2 patients [24]. As a result, at 15 years the overall survival rate for D2 patients improved (D1, 21%, D2, 29%) with lower instances of disease-related deaths (D1, 48%; D2, 37%) [24]. As de Bree et al. [25] indicates, high rates of morbidity and mortality attributed to surgery in the Dutch trial may have resulted from inadequate surgeon training, and a challenging learning curve associated with the procedure, in combination with low case numbers which may not have provided opportunities for the involved surgeons to maximise their competency during the trial. In addition, unlike in 1995 when the Dutch trial was performed, splenectomy and pancreatectomy are no longer routine for D2 lymphadenectomy. As such, with adequate surgeon training, performing a more targeted D2 lymphadenectomy (without splenectomy or pancreatectomy) reduces the morbidity and mortality associated with this procedure, while preserving the disease-related survival advantage of D2 [25, 26].

Critically, these analyses highlight the importance of an approach to gastric lymphadenectomy that comprehensively and thoroughly identifies and removes high-risk LNs, as well as the benefit of clear guidance during resection to assist surgeons.

It is within this paradigm that we believe a dual-modality magnetic/fluorescent approach to LN mapping offers advantages over conventional approaches. The present study investigated the pairing of FerroTrace and ICG for more accurate gastric SLN localization. This concept was initially tested using robot-assisted dissection (*n* = 2) given the promising results previously obtained with this tracer combination in the head and neck [17]. The robot-assisted investigation demonstrated concurrence of magnetic and fluorescent signals and the ability to use MRI to plan surgery, but was more akin to the conservative pick-up method of SLNB, as the draining lymphatic basin was only partially dissected. In the interest of fostering more extensive dissection in a more accessible manner (i.e. to easily locate and remove all ICG-positive LNs akin to the SNNS approach), the robot-assisted technique was replaced with handheld instruments and exploratory post mortem laparotomy (*n* = 6). In both instances, application of the dual-modality concept to gastric SLN localization demonstrated efficacy as a result of the multi-staged approach involving preoperative lymphatic mapping with MRI (to determine the location of magnetic LNs); intraoperative fluorescence-imaging guidance to clearly highlight draining lymphatics; and identification of the SLN among removed specimens via ex vivo magnetometer probe.

In a clinical setting, we anticipate preoperative MRI will help plan lymphadenectomy by clearly highlighting occurrences where draining (magnetic) LNs are located outside of conventional surgical fields—for example, if D1 lymphadenectomy is planned but magnetic LNs are found in D2 stations. In combination with intraoperative lymphatic mapping by fluorescent ICG, this approach can provide clear differentiation of the draining lymphatic structures, reduce intraoperative bleeding [7], and provide surgical guidance to enable less experienced surgeons perform more targeted lymphatic resections (i.e. SNNS), which potentially results in reduced patient morbidity and mortality. Following *en bloc* dissection of lymphatic tissue, ex vivo pathological examination of LNs using a magnetometer probe is anticipated to accurately identify individual SLNs within the excised specimen. This allows for a more focussed and detailed pathological assessment (i.e. serial sectioning and immunohistochemistry ‘ultra-staging’ of individual SLNs), positively impacting the accuracy of patient prognosis and helping clinicians make a more informed decision about the most appropriate treatment options. Indeed, ultra-staging in place of single sectioning and H&E stain to assess the tumour burden of LNs is expected to reduce false-negative staging rates and overcomes the limitations of simplified pathology as described in the Japanese JCOG0302 study [27].

While we are satisfied that the results of our study demonstrate preclinical efficacy of the dual-modality magnetic-fluorescent approach to SLN localization in the gastric environment, a complete transition to clinical application warrants comprehensive discussion about the strengths, limitations, and potential solutions to the challenges associated with this novel approach.

To begin with, the protocols developed for preoperative MRI have been optimised for the detection of FerroTrace particles, which are highly susceptible and produce strong levels of contrast to surrounding tissue, even at low concentrations. Overall, preoperative MRI detection of FerroTrace-positive nodes proved to be successful in all but one instance (94% success rate; *n* = 15), though at times ease of identification was dependent on animal anatomy (e.g. proximity of LNs to the large bowel). The T2 protocol used here was not difficult to implement, and involved relatively minor modification of an existing Siemens imaging protocol (2D GRE FLASH). The T1 VIBE protocol was based on a Siemens work in progress (WIP), so is not currently commercially available; however, this protocol was only relied upon in one instance (animal 5, as shown in Fig. 2) when the magnetic signal was partially obscured by the large bowel and cross-referencing between T2 axial and coronal planes was not sufficient to confirm the presence of FerroTrace. Given the reduction in MRI signal of FerroTrace that occurs from shortening TE in the T1 VIBE scan, future use of this protocol for combatting endogenous bowel contrast may only be feasible when FerroTrace uptake is at a sufficiently high level. Based on previous experience, the number of detectable magnetic LNs is not expected to increase beyond 6 hrs post-injection [16], however, the iron level and MRI signal strength continue to increase over time, so waiting longer than 1 h after injection to image gastric LNs may improve MRI detection rate. Fortunately this reflects a clinical reality, where FerroTrace would be administered in theatre, and several hours—or even days—may separate injection and MRI. Additionally, interfering sources of negative MRI contrast could be reduced if the injection is performed endoscopically (thus preventing the ingress of gas pockets prior to MRI), with recent evidence suggesting comparable outcomes between submucosal and subserosal injections of ICG [28]—though this is yet to be tested with FerroTrace. Where appropriate, prepping the bowel prior to the procedure might also help reduce confounding MRI artefacts in the GI tract.

**Fig. 5:**
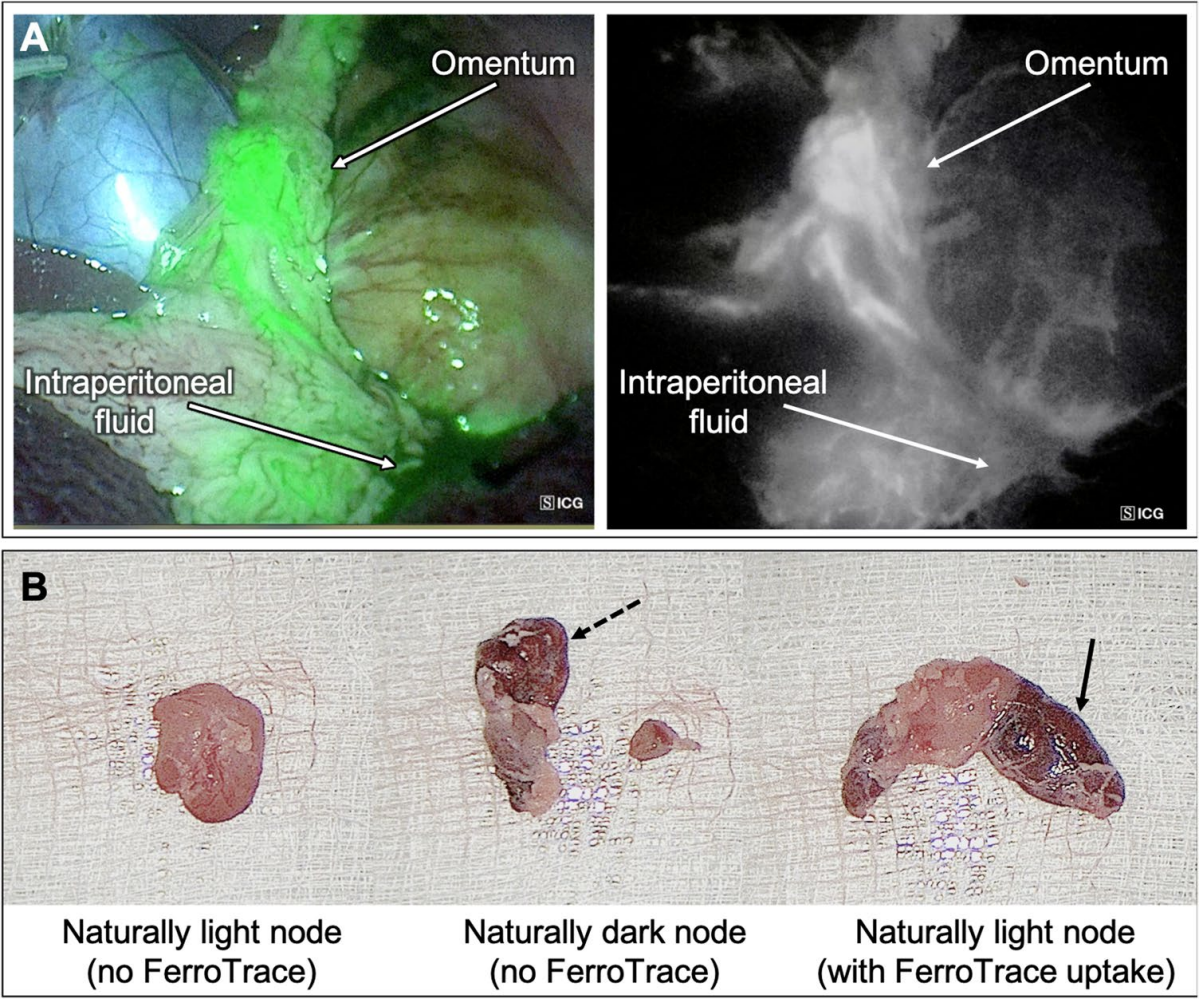
**A** Example of background fluorescence created by ICG dye leakage during the injection, particularly among the omentum and free intraperitoneal fluids. **B** Three examples of swine gastric lymph node discolouration; left, a naturally light-coloured node; middle, a naturally dark-coloured node; right, a naturally light-coloured node that has been discoloured due to the uptake of FerroTrace. Note the difficulty to clearly differentiate endogenous dark colouration (dashed arrow) from exogenous discolouration from FerroTrace particles (solid arrow) (Color figure online)

Secondly, as discussed previously, ICG has a low molecular weight and is thus transported rapidly through the lymphatics, washing through the SLN(s) and into higher echelon LNs within minutes. However, unlike visible dyes, ICG has the advantage of producing an infrared fluorescent signal that can penetrate short distances through perigastric and adipose tissues which improve visibility and detection [4, 29, 30] and cannot be confounded by the presence of other pigments such as those used to visualise tumour boundaries (e.g. India ink). In our dual-modality paradigm, it is accepted that the ICG will not be specific to the SLNs, and will likely highlight a relatively high number of LNs in a short period of time. However, ICG has proven effective at highlighting the pathways leading from the injection site to SLNs, and from SLNs to 2nd echelon LNs and beyond to guide dissection [17, 30]. During the laparotomy phase of this study, sensitive fluorescence imaging guidance yielded an additional advantage: rapid identification of draining LNs, with a mean time of 2.5 ± 1.7 min between starting the ICG mapping to first LN removal. Despite the advantages provided by fluorescence imaging, a practical limitation was observed as a result of the high sensitivity of fluorescence cameras: during dissection, small amounts of ICG leakage from the injection site may create a strong background signal if the dye spread and mixed with free intraperitoneal fluid (Fig. 5). Similarly, the greater omentum (caul fat) tended to superficially retain leaked ICG in a sponge-like manner, which could potentially obscure fluorescent vessels between the injection site and LNs. It was noted throughout the study that leakage tended to be minimal when a single bleb was successfully delivered to the submucosa, whereas the more superficial the injection (i.e. delivering some dose to the smooth muscle layer), the greater the leakage. During laparotomy, leaked dye was effectively cleaned up using abdominal sponges and by retracting the greater omentum, which greatly improved the ability to locate fluorescent lymphatic vessels and draining LNs.

In this study, we took advantage of the differences in pharmacokinetics of the two tracers to determine the relative LN specificity of the magnetic FerroTrace tracer to ICG—whereby higher LN specificity is synonymous with reduced flow-through. This is done by comparing the number of ICG-positive LNs (taken to be the complete drainage basin) to the number of FerroTrace-positive LNs. A mean number of 4.7 ± 1.2 ICG-positive LNs were removed per animal, compared to 2.0 ± 1.3 LNs containing FerroTrace (all of which were also ICG-positive). This demonstrates a statistically significant difference between LN specificity of the two tracers (*p *value = 0.0039). For FerroTrace, this reduced flow-through is the result of larger particle size compared to ICG, and the addition of the mannose label. The mannose label in particular is believed to produce the stratification of tracer distribution within draining nodes, with 76 ± 30 % of lymphotropic tracer collecting in the SLNs, equating to 88 ± 20% of magnetometer signal. Thus, probe detection provides a clear differentiation of SLNs from higher echelon LNs.

Finally, previous experience with FerroTrace has demonstrated that the dark brown colour resulting from the accumulation of the magnetic tracer in LNs means it can play a secondary-role as a lymphotropic dye. Due to the specificity of FerroTrace, this colouration can be used to differentiate SLNs from higher echelons (i.e. SLNs are typically darker in colour [16]). However, in the gastric model, endogenous colouration of LNs limits this secondary-role of FerroTrace as a visible dye, with some magnetic-negative LNs exhibiting a similar colouration to those containing FerroTrace (Fig. 5). As such, for this study the use of ICG fluorescence imaging was critical, and in instances where endogenous colouration prevented the visual identification of FerroTrace-positive nodes, the magnetometer probe was the most efficient means for confirming a magnetic signal. Though the magnetometer proved the least sensitive detection method compared to MRI or iron quantification (86% SLN detection rate; 75% overall detection rate), the limitations of the current embodiment of the FerroMag probe used here are well understood (short detection range, lower sensitivity compared to coil-based magnetometers) as discussed elsewhere [31]. Of the 3 false-negative LNs measured by the magnetometer, the mean FerroTrace-iron accumulation was 75 µg by ICP-MS (range 40–134); however as magnetometer signals are influenced by the size of the LN and the distribution of tracer within, this number alone may not be informative. Instead, the FerroTrace-iron concentration (iron quantity per gram of LN) may be a more useful metric, which in this study was 83 µg iron per LN-gram (range 50–122). For future clinical application a more sensitive magnetometer probe would be required to improve detection accuracy. If laparotomy is performed, such a handheld probe may be used intraoperatively along with fluorescence-imaging to help guide SLN localization. For laparoscopic or robotic-assisted procedures, ‘drop-in’ magnetometer probes capable of fitting through trocar ports are currently being developed [32, 33] and would provide a less invasive means for intraoperative magnetometer detection. Such probes are not currently commercially available, and there exist several non-trivial technical hurdles that have to be overcome—for example, reducing the probe size to fit through a trocar port greatly reduces magnetometer sensitivity, and metal laparoscopic or robotic instruments can induce spurious magnetometer signals. Nevertheless, advancements in magnetometer technology in this direction would allow for intraoperative identification of the SLNs prior to dissection, which could be expected to improve surgeon confidence that the correct lymphatic regions are being dissected.

While the present study was focussed on the efficacy of the dual-modality approach for LN mapping, discussion of how this approach can be integrated with neoadjuvant therapy (NAT) is warranted. NAT is commonly used in patients with locally advanced gastric cancer, whereby chemotherapy and/or chemoradiotherapy, potentially combined with targeted therapy and/or immunotherapy, is administered prior to surgical resection. In these patients, NAT is linked to improved survival outcomes [34]. Postoperatively, the regression rate of LNs can be graded by pathology to assess the success of NAT and determine if further interventions (e.g. additional chemotherapy) are required [35]. Recent evidence has indeed demonstrated that improved disease-free survival correlates with LN regression rate [36], placing a prognostic burden on collection and analysis of the correct LNs.

Though for our dual-modality approach the use of ICG is relegated to intraoperative guidance, the use of FerroTrace and preoperative MRI may provide useful additions for patients undergoing NAT. Current NCCN guidelines recommend preoperative laparoscopic staging for all gastric cancer patients planning to undergo NAT [37], hence for these patients FerroTrace could be injected during this scheduled laparoscopy, reducing the number of procedures required prior to lymphadenectomy. Following FerroTrace injection, high resolution MRI could utilise the magnetic signal of draining LNs to enhance guidance for targeting radiotherapy. The administration of FerroTrace prior to NAT is possible due to the excellent long-term retention in draining LNs [16]. Here, the combination of magnetic ‘tagging’ of LNs with ex vivo magnetometer detection could facilitate the identification of prognostically significant LNs used in regression rate grading.

Despite these possible advantages, there are still several questions around the impact of NAT on the ability of FerroTrace and/or ICG to accurately map the draining lymphatics at the time of surgery. Such questions include: Does FerroTrace remain in the LNs during NAT, or do interventions such as chemoradiotherapy affect the retention and distribution of FerroTrace in draining LNs? Is a second injection of FerroTrace required at the time of surgery (e.g. to ‘top-up’ the SLN such that the magnetometer can still identify it from higher echelon LNs)? Is there still concurrence between pre-NAT FerroTrace and post-NAT ICG uptake (e.g. have the lymphatic pathways changed as a result of NAT)? The present study is unable to answer these questions, but they are critical to understanding the clinical translation of the proposed dual-modality lymphatic mapping approach. Future works planned include phase 1 clinical trials in gastric cancer, which would involve comparing the efficacy of the dual-modality approach in NAT and non-NAT patient cohorts in an attempt to answer such questions. Such a trial would also be used to better understand the cost-benefit outcomes of adding FerroTrace as an additional tracer to be used with ICG.

The findings of this preclinical investigation are that the dual-modality FerroTrace-ICG approach to gastric SLN localization is feasible and may result in more accurate identification of SLN(s) as a result of: preoperative planning via MRI; highly sensitive lymphatic mapping via ICG fluorescence; and by the high specificity of FerroTrace to SLNs which facilitates their detection via magnetometer probe. In addition to providing surgical guidance for dissection, we anticipate the use of FerroTrace could play a role in the complete treatment paradigm for locally advanced gastric cancers, which would include preoperative NAT planning and postoperative regression rate grading of magnetic LNs.
